# Influence of ROI selection on resting state functional connectivity: an individualized approach for resting state fMRI analysis

**DOI:** 10.3389/fnins.2015.00280

**Published:** 2015-08-11

**Authors:** William S. Sohn, Kwangsun Yoo, Young-Beom Lee, Sang W. Seo, Duk L. Na, Yong Jeong

**Affiliations:** ^1^Department of Bio and Brain Engineering, KAISTDaejeon, South Korea; ^2^Department of Neurology, Samsung Medical Center, Sungkyunkwan UniversitySeoul, South Korea; ^3^Neuroscience Center, Samsung Medical CenterSeoul, South Korea

**Keywords:** subject-specific ROIs, resting state fMRI, individual variability, graph theory, aging

## Abstract

The differences in how our brain is connected are often thought to reflect the differences in our individual personalities and cognitive abilities. Individual differences in brain connectivity has long been recognized in the neuroscience community however it has yet to manifest itself in the methodology of resting state analysis. This is evident as previous studies use the same region of interest (ROIs) for all subjects. In this paper we demonstrate that the use of ROIs which are standardized across individuals leads to inaccurate calculations of functional connectivity. We also show that this problem can be addressed by taking an individualized approach by using subject-specific ROIs. Finally we show that ROI selection can affect the way we interpret our data by showing different changes in functional connectivity with aging.

## Introduction

An important aspect of network analysis and graph theory in brain imaging is node definition (Smith et al., 2011; Shen et al., 2013). These nodes represent neural populations in the brain which have shared structural or functional relevance (Sporns et al., 2004; Bullmore and Sporns, 2009). Nodes that share a common functional purpose are connected among one another to form a network. Recently, studies have utilized resting state functional magnetic resonance imaging (fMRI) to obtain functional network properties of the whole brain (Salvador et al., 2005; He et al., 2009; Wang et al., 2010). These studies have shown that fluctuations in the spontaneous blood oxygen level dependent (BOLD) signal exhibit a high degree of correlation between regions with known functional similarities (Biswal et al., 1995; Damoiseaux et al., 2006; Raichle, 2011). Therefore, resting state fMRI is able to map functional connectivity in the absence of any overt task (task-free) during the process of image acquisition. The simplicity in its design, image acquisition and analysis has made resting state analysis popular in fMRI studies. Resting state analysis has been useful in examining changes in brain connectivity with age (Stevens et al., 2009; Supekar et al., 2009; Ferreira and Busatto, 2013). The human lifespan is characterized by the initial development and later decline of cognitive abilities from adolescence through aging. These changes in our cognitive abilities are thought to be representative of changes in the functional organization of our brains.

In graph theory, nodes are created using a seed-based approach in resting state analysis. Regions of interest (ROIs) in the brain are selected, and the time-series of the BOLD response is extracted to represent the resting state neuronal activity (Fox and Raichle, 2007). The extracted time-series are then correlated with one another to determine connectivity among the different nodes. The methods and parameters of ROI selection are often different across studies (Poldrack, 2007). Consequently, with resting state analysis, there is no established standard for ROI selection. ROIs are usually selected based on brain atlases, group independent component analysis (ICA), or previous functional studies (Cole et al., 2010). Additionally, in resting state fMRI, it is common to use the same ROIs for all subjects in a given study. This poses a problem because using ROIs that are the same, or standardized across subjects, ignores the issue of subject variability. While the brain, for the most part, shares common functional topographies across subjects, they are not identical for every person (Mohr and Nagel, 2010; Mueller et al., 2013). Thus, while a specific region of the brain might be a good representation of a functional region for one individual, it is highly probable that it will be misrepresentative for another. As such, it becomes debatable whether the extracted time series from a given ROI is the actual desired signal of interest (Shen et al., 2013).

We propose that using the same ROIs for all subjects is fundamentally flawed because it assumes spatial similarity between subjects. In addition, since these ROIs can be inaccurate representations of the desired signal of interest, the measures of connectivity that result from these ROIs can be inaccurate as well. In this study we will show the differences in calculated functional connectivity using different ROI selection methods. We show that methods which use a standardized set of ROIs for all individuals result in lower calculated functional connectivity and higher variance than an individualized approach. Finally, we show that this process of ROI selection could affect the way data could be interpreted in aging which can result in entirely different conclusions. From the results of our study we propose that ROI selection should be conducted individually for a more accurate analysis of resting state functional connectivity.

## Methods

### Subject demographics

Twenty-two right handed young adults, ages 20–32 years old, and 20 elderly cognitive normal subjects, ages 65–80 years old underwent 5 min resting state fMRI scans. Data was acquired at Samsung Medical Center and this study was approved by the Institutional Review Board at Samsung Medical Center. All participants and/or caregivers provided written, informed consent for participation in this study.

### MRI acquisition

A 3.0 Tesla scanner (Model: Philips Intera Achieva, Phillips Healthcare, The Netherlands) was used for resting state MRI acquisition. Participants were instructed to lay motionless with their eyes open during image acquisition. The scans involved the acquisition of 35 axial slices using a gradient echo planar imaging pulse sequence: voxel size (RL, AP) = 2.875 × 2.875 mm with a slice thickness of 4 mm. TE = 35 ms; TR = 3000 ms; FOV (RL, AP, FH) = 220 × 140 × 220 mm. T1-weighted anatomical images were obtained for each participant (TE = 10 ms; TR = 1114 ms; FOV (RL, AP, FH) = 220 × 220 × 132 mm, REC voxel size = 0.43 × 0.43 × 0.43 mm).

### Pre-processing

Pre-processing of the resting state fMRI and structural MRI data was performed using MRIcron (http://www.cabiatl.com/mricro/mricron/index.html) and the FMRIB Software Library (FSL, www.fmrib.ox.ac.uk/fsl/). MRIcron converted the raw fMRI images to a compressed FSL format. Image pre-processing consisted of skull stripping using the Brain Extraction Tool (BET), splice timing correction, temporal high-pass filter (Gaussian-weighted least-squares line fitted with sigma = 100.0 s), MCFLIRT motion correction and spatial smoothing (using a Gaussian kernel of FWHM 4 mm). FLIRT (FMRIB's Linear Image Registration Tool) was used to register and normalize the images to the Montreal Neurological Institute (MNI) template (2-mm resolution). Group measures of head movement was measured using the FSL Motion Outliers with framewise displacement (FD). Additional motion correction steps and nuisance regression was not performed. For validation purposes, white matter and CSF signals are identified from ICA and regressed out using partial correlation.

### Comparison of ROI selection methods

**Figure 1:**
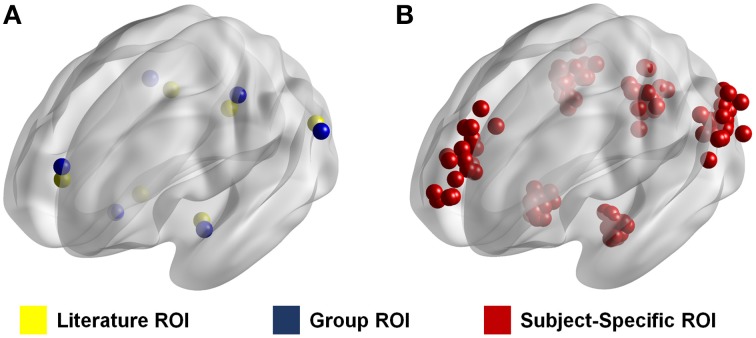
**ROI location from three different methods**. Figure shows the location of each ROI for each method. Literature ROIs are shown in yellow and group ROIs are shown in blue **(A)**. In these two methods the ROIs are standardized such that they are the same for every individual. Subject-specific ROIs are shown in red **(B)**. In this method the ROIs are different for every subject as represented by the many ROIs in the figure. Each dot represents an ROI in a given node for one specific individual.

To show the effects of ROI selection on the calculated resting state connectivity, we calculated the intrinsic connectivity of the anterior default mode network (aDMN) using three different methods for ROI selection (Supplementary Figure 1). The first two methods each use a conventional set of ROIs in which each set is standardized across subjects. In the first method, ROIs were determined from the results of a previous study that looked at the connectivity of the DMN (Watanabe et al., 2013). These ROIs will be referred to as “literature ROIs” (Figure 1). The second method used group independent component analysis (ICA) using the Multivariate Exploratory Linear Optimized Decomposition into Independent Components (MELODIC) toolbox in FSL (Beckmann and Smith, 2005). Group ICA was run on the young subject group with output 30 components. The seed coordinates were determined by the peak z-values in the spatial maps of the targeted functional networks. Since these ROIs were generated based on the data used in the study, these ROIs will be referred to as “Group ROIs” (Figure 1). In the third method, resting state networks for each individual subject were reconstructed in a manner similar to the dual-regression method (Filippini et al., 2009). However, in this case, instead of spatially regressing the ICA spatial map, the time-series which was associated with the target network was regressed directly in each subject for network reconstruction. In other words, the associated time-series for a network obtained in ICA is used to reconstruct that network for a specific individual. To do this, we took the time component of each target ICA network and identified the time-series for each individual. These time-series were then mapped individually using the FEAT toolbox in FSL (Smith et al., 2004; Jenkinson et al., 2012). Seed coordinates were determined by the peak z-values in the reconstructed networks for each individual, which resulted in a unique set of ROIs for each subject. These ROIs will be referred to as “subject-specific ROIs” (Figure 1).

When seeding the aDMN, 6 × 6 × 6 mm cubic ROIs were drawn in each of the major node regions. These regions included the left and right lateral parietal lobe (PLL, PLR), the posterior cingulate cortex (PCC), the dorsal prefrontal cortex (dPFC), and the left and right hippocampus (HCL, HCR).

To determine the effects that spatial location had on calculated connectivity we calculate the average z-stat a given ROI had in the reconstructed aDMN network for each individual. This information gave the correlation to the aDMN of a region of the brain that would be seeded using various methods. Low z-stats indicated that the region of the brain seeded had a low correlation with the aDMN, and high correlations indicated high correlations with the aDMN for that individual. In essence, a low average z-stat showed the incorrect seeding of the aDMN and vice versa. This average z-stat was correlated with average calculated connectivity with other nodes to show the effect that incorrect seeding has on calculated connectivity.

Connectivity between ROIs was obtained by extracting the average time-series from each ROI and calculating the correlation using Pearson's correlation coefficient. Statistical significances between methods of ROI selection were calculated using paired *t*-tests. Visualization of ROIs and brain networks was performed using the BrainNet software (Xia et al., 2013).

### Graph theory analysis: aging effects

The accurate calculation of functional connectivity can have significant implications in data analysis and interpretation. A major area of interest in neuroscience is the study of how functional connectivity in the brain changes with aging. To examine changes during aging, an additional elderly subject group of 20 subjects with no history of psychological illness was included. Furthermore, to examine changes in major brain network topographies, four additional networks were included for analysis (Supplementary Figure 1). These networks included the posterior DMN (pDMN), the left and right frontoparietal network (FPNL, FPNR), and the salience network (SAL). For each network, one seed region was created for each major node. Four regions were seeded for the pDMN: the PCC, PFC, PLL, and PLR. Seven regions each were seeded for both the FPNL and FPNR: the PCC, ACC, left and right inferior parietal sulcus (IPSL, IPSR), left and right inferior frontal gyrus (IFGL, IFGR), and the respective occipital temporal cortex (OTC). Finally five regions of the SAL network were chosen for seeding: the ACC, the left, and right prefrontal cortex (PFCL, PFCR), and the left and right insula (IL, IR). When seeding each region, 6 × 6 × 6 mm cubic ROIs were used. A total of 30 seeds were used for analysis.

To show how ROI selection can affect data interpretation, three different methods for seed selection were used. The first two methods used group ROIs and the third method used subject-specific ROIs. The first two group ROIs were generated based on the different subject groups; the first group ROI set was derived from the young subject group and the second group ROI set was derived from the old group. In other words, ICA analysis was performed on both the young and old subject groups separately. One set of ROIs was generated from the results of each ICA analysis, resulting in a young subject or “young group ROI” set and an elderly subject or “old group ROI” set. Subject-specific ROIs were derived in the same way as the first section of our analysis. Individual networks were reconstructed from ICA to identify peak voxels within each major node in the various networks for seeding. Time series are extracted from each ROI and correlated with each other to obtain resting connectivity.

Connectivity between ROIs was obtained using a Pearson's correlation coefficient between the extracted time series from each ROI to create a set correlation matrices for each method in every subject. This results in every individual having three correlation matrices, one calculated from ROIs derived from young subjects, one from ROIs derived from elderly subjects, and finally one derived from subject specific ROIs. Individual correlation matrices are thresholded at *r* > 0.35 to create binary matrices for graph theory analysis. The threshold was set high intentionally so that only high correlation edges were considered. Networks representations were constructed from averaged correlation matrices for each group which are converted to binary matrices using a threshold of *r* > 0.35. Network visualization was done using Pajek—Program for Large Network Analysis (http://pajek.imfm.si/doku.php?id=pajek).

Significant differences between old and young groups were calculated using unpaired *t*-tests. Bonferroni correction was used for multiple hypothesis testing (α/435). Network properties were calculated for each individual using Matlab scripts from the Brain Connectivity Toolbox (BCT) (Rubinov and Sporns, 2010) and Matlab Tools for Network Analysis (http://strategic.mit.edu/downloads.php?page=matlab_networks). Properties were calculated using binary undirected matrices. Modularity was calculated using the Louvain method for community detection.

## Results

### Resting state correlation analysis

**Figure 2:**
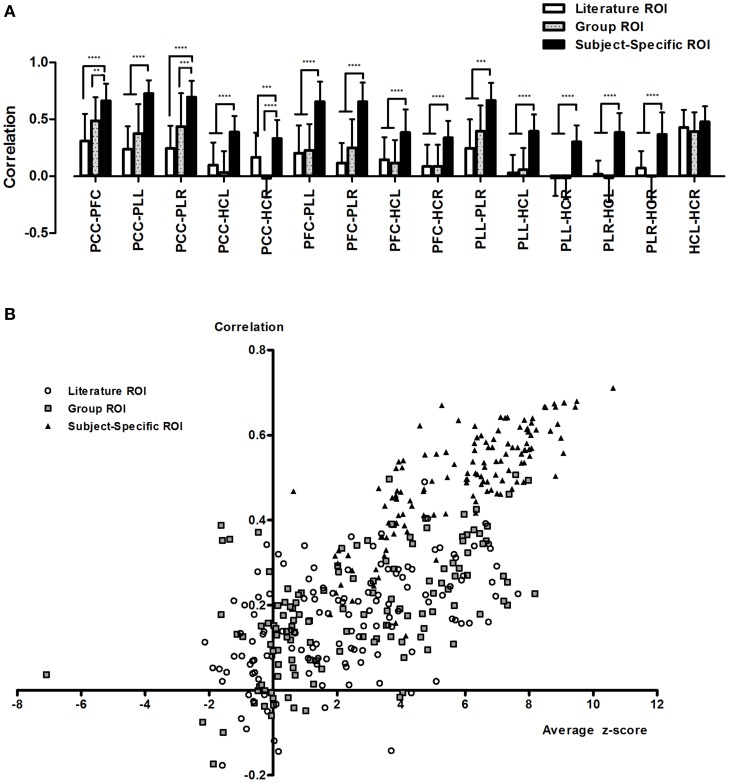
**Calculated resting state functional connectivity differs depending on the method of ROI selection**. Calculated resting connectivity for literature ROIs, group ROIs and subject-specific ROIs **(A)**. In addition to higher correlation, subject-specific ROIs showed lower variance. This can be attributed to incorrect seeding with literature and group ROIs **(B)**. ^**^p < 0.01; ^***^p < 0.001; and ^****^p < 0.0001.

Analysis of different methods of ROI selection showed that ROI selection has a drastic effect on the calculation of resting state connectivity. Overall, the average connectivity between nodes was significantly higher when using subject-specific ROIs compared to the other sets of ROIs (Figure 2A, Supplementary Figures 2A–C). The general trend showed that the more relevant a set of ROIs were to the data set, the higher the calculated connectivity. For the most part, subject-specific ROIs showed significantly higher correlations (*p* < 0.0001) compared to literature and group based ROIs. In addition, literature and group ROIs have higher variance than subject specific ROIs (Figure 2A, Supplementary Figures 2D–F).

Calculations of average ROI z-stats showed that the literature and group ROIs exhibited consistently low z-stats compared to subject-specific ROIs (Figure 2B). In addition, if we were to arbitrarily threshold our image at *z* > 2.3 (a commonly used threshold in fMRI studies), we would find that for the majority of subjects, the literature and group ROIs will not fall in the clusters of aDMN for more than half the ROIs. On the other hand, we found that the majority of ROIs using a subject-specific approach fell consistently within the aDMN. As a result, we found that average calculated connectivity is lower when ROIs fell outside the aDMN and high when the ROIs are inside the aDMN (Figure 2B).

### Graph theory analysis: aging effects

**Figure 3:**
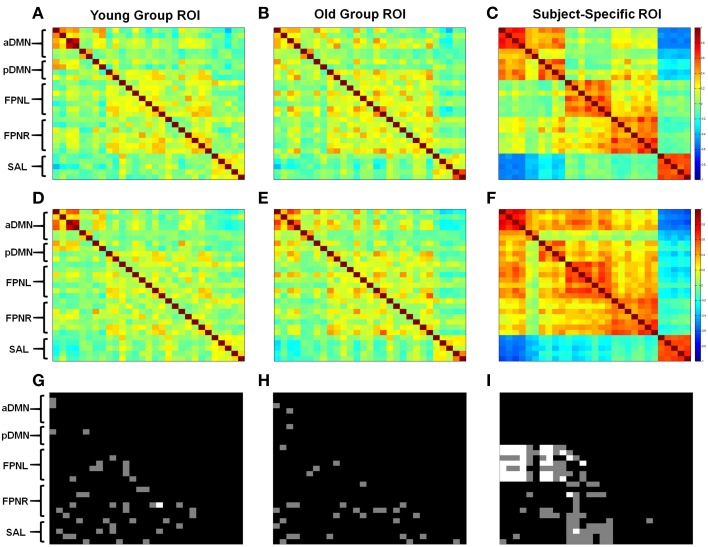
**Different methods of ROI selection changes how changes in connectivity with aging are interpreted**. Calculated correlation shows different changes in functional connectivity. Obtained correlation reveals different connectivity with young **(A–C)** and old **(D–F)** subjects. Statistical differences are shown between old and young subjects using young group ROIs **(G)**, old group ROIs **(H)**, and subjects-specific ROIs **(I)**. Gray regions show statistical differences for *p* < 0.05 and white regions show statistical difference for *p*_*Corrected*_ < 0.05. ROIs are listed from top to bottom for each network. aDMN: PCC, PFC, PLL, PLR, HCL, HCR. pDMN: PCC, PFC, PLL, PLR. FPNL: ACC, PCC, IPSL, IPSR, IFSL, IFSR, OTC. FPNR: ACC, PCC, IPSL, IPSR, IFSL, IFSR, OTC. SAL: ACC, PFCL, PFCR, IL, IR.

Correlation analysis again showed lower connectivity with the group based methods compared to subject-specific ROIs (Figure 3). Significant changes in connectivity with aging differed depending on the ROI selection method. With young subject group ROIs we observed increases and decreases in network connectivity with aging. However, most importantly we saw a decrease in connectivity in the aDMN and an overall breakdown in network connectivity (Figure 3G). With the old subject group ROIs, again we observed both increases and decreases in connectivity; however, when looking at the aDMN, we observed a significant increase in connectivity in addition to the formation of connectivity between nodes (Figure 3H). These results contrast with those derived from the young group ROIs. Finally, subject-specific ROIs generally showed no significant changes in intrinsic network connectivity with aging, with the exception of the FPNL (Figure 3I). However, significant changes in between network connectivity was more apparent particularly in the left and right FPN (Figure 3I). With aging, the FPNL and FPNR showed significantly increased connectivity with both the aDMN and pDMN. In addition the FPNL and FPNR showed a loss of connectivity or a formation of anti-correlation with the SAL network.

**Figure 4:**
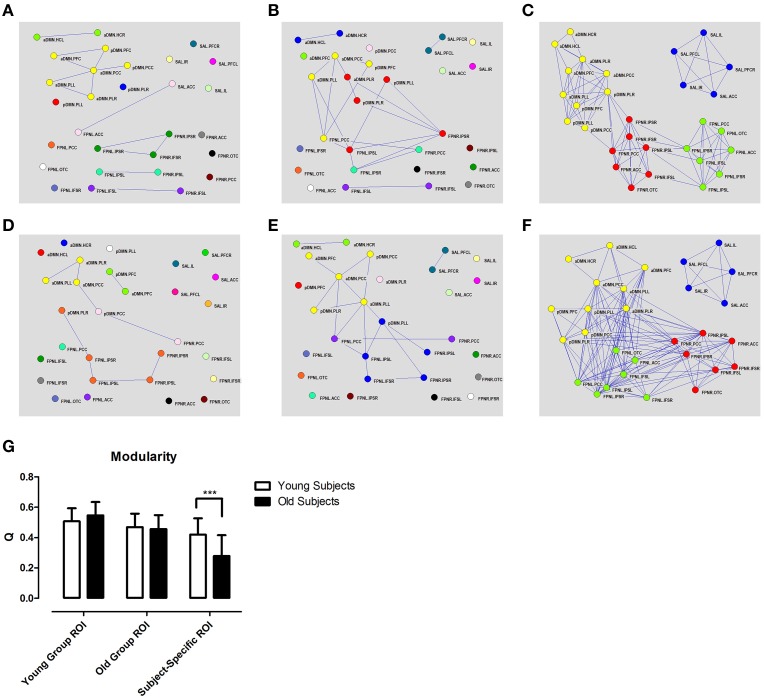
**Different methods of ROI selection changes how changes in connectivity with aging are interpreted**. Different calculated correlation and topographical organizations of the brain in young **(A–C)** and old **(D–F)** subjects (*r* > 0.35). High correlation among regions of the DMN (shown in yellow) are only observed if the ROIs are derived from that specific group. Graph theory analysis shows decreased in modularity using subjects-specific ROIs **(G)**. Different colors represent different networks as determined from modularity analysis. Subject specific ROIs show consistent partitioning into 4 distinct networks in both old and young subjects. ^***^p < 0.001.

Graph network construction showed little or no connectivity between networks using the group based ROIs (Figure 4). Particularly, in the aDMN, we could observe a loss of connectivity with the young group ROIs (Figures 4A,D) but not with the old group ROIs (Figures 4B,E). In the case of subject-specific ROIs, there were no real changes in intrinsic connectivity; however, there was an increase in between network connectivity which, resulted in a loss of modularity (Figures 4C,F). Various graph theory properties which were measured included clustering coefficient, modularity, assortivity, and efficiency. Analysis of these different properties revealed no real differences with aging except for modularity when using the subject-specific ROI approach (Figure 4G, Supplementary Figure 3).

Connectivity between the aDMN and pDMN showed practically no connectivity with group based methods (Figures 3A,B,D,E). However, subject-specific ROIs showed a high degree of connectivity between the two networks (Figures 3C,F). The changes in connectivity are also the same with aging. Both networks show a significant increase in correlation to the FPNL (Figure 3I). Finally, graph theory analysis reveals that the two networks are hardly connected with group based methods (Figures 4A,B,D,E). Subject-specific ROIs on the other hand, show the two networks as part of the same module, with high connectivity between nodes (Figures 4C,F).

## Discussion

Currently, neuroimaging studies have largely ignored or marginalized the issue of subject variability. While major brain networks exhibit similar spatial characteristics between individuals, they are not exactly the same. Therefore, a generalized map of the human connectome can, at best, provide only a rough reference for major brain connections (Lichtman and Sanes, 2008).

We found that by using subject-specific ROIs, we were able to obtain higher correlations and lower variance compared to using ROIs that were based on group averages or previous research (Figure 2A, Supplementary Figure 2). Intuitively, higher correlations between nodes of the same network provide a more accurate representation of network connectivity, since brain networks are by definition, regions of the brain that exhibit a high degree of correlation with one another (Fox and Raichle, 2007; Bullmore and Sporns, 2009). Lower connectivity obtained using ROIs that are standardized across individuals could be attributed to subject variability since the exact location of a major brain network node is not the same from person to person (Mueller et al., 2013). As a result, the use of incorrect ROIs will result in lower calculated connectivity and higher variance as the ROIs used will “miss” the correct node location for certain individuals (Figure 2A). When examining the spatial locations of each ROI with relation to the DMN for each individual, we find that for literature and group ROIs there is a high chance that they will not fall in the DMN for a given individual (Figure 2B). This means that, for those individuals, the calculated correlations obtained are not the real correlations between regions of the DMN. This is extremely important in graph theory because the calculated connectivity between nodes is often used to determine whether or not an edge exists. This can have huge ramifications in interpreting data. For example, analysis with literature ROIs yielded the result in our data that DMN connectivity was all but non-existent while subject-specific ROIs showed strong DMN connectivity. Thus, while we may have defined a standard set of ROIs that was used across individuals as part of the same network (literature and group ROIs), the low calculated connectivity suggests that these ROIs are incorrect for a majority of the subjects. This issue can be compensated somewhat by enlarging the size of the ROI (Supplementary Figure 4), which increases the likelihood that the brain region representing that particular individual's node will be included. However, this also increases the likelihood of including voxels that are not part of the desired target network at all, causing a decrease in signal to noise ratio. Thus, the extracted time signal will not be a true representation of the node of interest.

Accurate computation of brain networks is essential because incorrect node definition can lead to misrepresentations of the functional changes which can occur across different cognitive states or disease. It is well-established that the DMN shows decreased correlation or deterioration with aging (Koch et al., 2010; Tomasi and Volkow, 2012; Vidal-Pineiro et al., 2014). However, these previous studies often used a seed based method, in which the ROIs were derived from young subjects. In this study, we replicated the findings of decreased connectivity within the DMN using ROIs based on our young subject group (Figures 4A,D). However, when the ROIs were derived from our old subject group, we observed increased DMN connectivity with age, which is contrary to previous literature (Figures 4B,E). These results show that ROI selection can have a direct effect on calculated connectivity by being biased toward the data from which they were derived. In comparison, the subject-specific ROIs showed no decrease or increase in correlation between regions of the DMN with aging (Figures 3C,F). Instead of network deterioration, the subject-specific ROI approach shows that between network connectivity is more affected. In this case, connectivity between the FPNL and the DMN and between the FPNL and the SAL increased significantly. This resulted in a loss of modularity with aging (Figure 4G). These results are in agreement with two previous studies which report loss of modularity and segregation between networks (Betzel et al., 2014; Chan et al., 2014) with aging. Finally, most convincing is that the majority of significant changes that were found with standardized ROIs did not survive multiple comparison testing (Bonferroni). However, with subject-specific ROIs a large portion of connections were significant, particularly connections between the FPNL and regions of the DMN.

The fractionation of the DMN into sub-networks is a commonly reported phenomenon in many studies (Andrews-Hanna et al., 2010; Jones et al., 2011; Van Oort et al., 2014). Therefore, while we have divided the DMN into two separate networks (aDMN, pDMN) for our analysis, they are in actuality two sub-components of the same network. Interestingly, if we look at the analysis using different ROI selection techniques, we find that standardized ROIs show low connectivity between node regions of the aDMN and pDMN (Figures 3, 4). In contrast, analysis of subject-specific ROIs shows high correlation between node regions between both networks (Figures 3, 4). Modularity analysis showed that both the aDMN and pDMN are part of the same module with subject-specific ROIs and are divided into many different modules with standardized ROIs (Figure 4). If we examine the changes in correlation with aging, we find that there is no pattern of change with standardized ROIs, however, with subject-specific ROIs, both the aDMN and pDMN showed similar changes (Figure 3I). In all these aspects, ROIs which are standardized across subjects, showed that the aDMN and pDMN were different networks, while subject-specific ROIs identified to two as part of the same overall network. When we examine other networks, we again see that only subject specific ROIs were successful in grouping ROIs into their respective networks (Figure 4). This supports the argument the subject-specific ROIs are needed for accurate calculations of functional connectivity.

The issue that methods of analysis can have an impact on results has been mainly investigated in the preprocessing stage (Aurich et al., 2015; Power et al., 2015). Particularly, studies in motion correction reveal that the breakdown in long range connections in aging could be attributed to motion (Power et al., 2012). The results of our analysis also show that it is entirely possible for the results of previous papers to be the results of analysis methods rather than actual changes that occur in aging. It should be noted that multivariate and voxel wide approaches, such as ICA and seed based voxel wide approaches, show decreases in correlation of the DMN with aging (Damoiseaux et al., 2008; Ferreira and Busatto, 2013). The simplest explanation for this is that, while the hub central regions of these networks do not experience a decrease in connectivity, the spatial extent or surrounding regions may be affected by aging. However, it also possible that these results may have been over interpreted. Studies have shown that with aging and in other neurological disorders individual differences increase (Macdonald et al., 2006). These “decreases” in connectivity observed in previous studies could merely be a result of larger differences within the elderly group or the younger group being more homogenous. If we follow that our connectome develops and changes with time, the exact spatial organization of our DMN network will also change with aging. Thus, while connectivity between regions of the DMN will not change, the spatial organization will (i.e., no change in intrinsic connectivity; however, there is network reorganization). This may have resulted in the observed decreases in certain voxels which were common in the younger age group, but, due to this reorganization, these voxels were no longer a part of the DMN in many elderly subjects. This is apparent in other studies which showed decreased connectivity using a voxel wise approach but showed no significant changes in calculated correlations between regions of the DMN (Koch et al., 2010). Further, studies and tests should be performed to resolve these discrepancies.

The results of our study and the significance levels were obtained without the use of preprocessing methods such as motion correction and nuisance regression. Supplementary Figure 5 shows the changes in calculated connectivity of the aDMN after WM and CSF regression. There effects on subject-specific ROIs were small, however with group ROIs, there was an increase in connectivity of the left and right hippocampus to other node regions and decreased connectivity between each other. This may be because of the spatial location of the hippocampus. Incorrect seeding of these regions may result in seeding regions with high WM or CSF signals. Therefore, an addition advantage in using subject-specific ROIs is that they are less susceptible to WM and CSF noise. Small ROIs derived using single-subject network reconstruction from ICA, allows for targeted extraction of functional time-series. Since the time series extracted are actually from functionally relevant regions from that particular individual, there is little need for nuisance regression. Motion analysis showed no significant different in head motion between young and elderly subjects (Supplementary Table 1).

Currently different automated parcellation methods have become popular methods of creating ROIs for graph theory analysis. These methods are successful in creating generalized maps of functional organization in the brain (Cohen et al., 2008; Power et al., 2011; Craddock et al., 2012). However, despite their success and popularity, many studies have shown different results in brain connectivity based on different parcellation methods which brings even more ambiguity as to which methods are more appropriate for resting state ROI selection (Wang et al., 2009; Van Den Heuvel and Sporns, 2011; Shirer et al., 2012; De Reus and Van Den Heuvel, 2013; Thirion et al., 2014). The use of subject-specific ROI proves an alternative to functional parcellation providing a solid method to define functional regions of the brain for graph theory analysis.

It should be noted that this study is not the first to explore the concept of different ROI selection strategies or analysis methods (Koch et al., 2010; Marrelec and Fransson, 2011). While these studies show slight differences between each analysis, the general conclusion is that the overall results are unchanged. Neither, is this the first study to utilize subject-specific ROIs in fMRI analysis (Golestani and Goodyear, 2011; Marrelec and Fransson, 2011). Many studies create subject-specific ROIs from task activation paradigms (Fedorenko et al., 2010; Weeda et al., 2011). However, since resting state fMRI lacks these task activation trials, this option is not available. Methods using dual-regression have begun to be a popular tool for analysis, however, despite individual reconstruction, these studies have failed to consider each data uniquely (Rytty et al., 2013; Smith et al., 2014). One study group created subject-specific ROIs from resting state networks similar to our study (Marrelec and Fransson, 2011). However, their results found no differences between using traditional standardized ROIs and subject specific ROIs. A major difference in our study is that the sizes of ROIs are much smaller. In the case of the other study they used a 12 mm sphere (6 mm diameter), which is a large ROI. When ROIs become this large, the different ROIs begin to overlap with one another which results in the similar calculated correlations which they obtain in their study. In fact when ROIs become rather large, the calculated connectivity can be very similar regardless of the method of ROI selection (Supplementary Figure 4D).

One drawback to this approach is that we can only perform analysis on functional regions which can be identified by ICA. Since traditional decompositions are only able to identify major brain networks (Beckmann et al., 2005; Damoiseaux et al., 2006; Ray et al., 2013), seeding of smaller subnetwork are more difficult. Identification of smaller subnetworks can be performed using high order ICA (Abou Elseoud et al., 2011), or localized ICA (Sohn et al., 2012; Beissner et al., 2014; Igelstrom et al., 2015). However, even in these cases it requires some a priori knowledge to identify functional regions for analysis.

The novelty of this paper is that it is the first to show that differences in calculated correlations with different ROI selection methods can have huge implications in the way data are interpreted. We propose that using small subject-specific ROIs located in each individual's network node regions provides a better representation of intrinsic network connectivity. Analysis using this method offers more insight into actual network connectivity than large bulky ROIs which more often than not include voxels which are not relevant to the signal of interest. In addition we show that by taking a more individualized approach, we are able to observe results which are absent in traditional approaches, such as the lack of change in DMN intrinsic connectivity with aging.

The brain is a complex system composed of different structurally and functionally interlinked regions. To map the functional connectivity between regions, an accurate definition of nodes is required. Whether, it is performed by parcellation, or seeding functional regions, the main issue of subject variability has been yet to be sufficiently addressed. More than anything this study aims to advocate an individualized approach to resting state fMRI by highlighting the advantages and dangers when using subject-specific ROIs and traditional approaches.

### Conflict of interest statement

The authors declare that the research was conducted in the absence of any commercial or financial relationships that could be construed as a potential conflict of interest.

## Supplementary material

Supplementary Figure 1 **Major resting state networks**. Networks were obtained using ICA. A total of five networks were selected for subsequent analysis.

Supplementary Figure 2 **Calculated resting state functional connectivity and variance from different ROI seeding methods**. Figure shows correlation values for each ROI selection method **(A–C)** and the variance **(D–F)**.

Supplementary Figure 3 **Graph theory properties for different ROI selection methods with aging**. Properties analyzed include assoritivity **(A)**, efficiency **(B)**, clustering coefficient **(C)**, and modularity **(D)**. Only the modularity obtained when using subject specific ROIs showed any significant differences in aging.

Supplementary Figure 4 **Calculated Functional Connectivity with respect to ROI size**. Graph shows how functional connectivity between nodes is affected when different ROI sizes are used for node selection when ROIs are selected from literature **(A)**, data **(B)**, and obtained for subject specific ROIs **(C)**. ROIs used are cubic ROIs and label denotes the length of a ROIs edge. Therefore actual ROI size will be n^∧^3 where n is the length of an edge. **(D)** Shows the calculated connected between nodes for different ROI selections methods using a 9 Voxel size ROI. PCC, posterior cingulate cortex; PFC, prefrontal cortex; LPL, left parietal lobe; RPL, right parietal lobe.

Supplementary Figure 5 **Calculated Functional Connectivity with WM and CSF regression**. Figure shows average connectivity of the aDMN using group ROIs **(A,C)** and subject-specific ROIs **(B,D)** when regressing WM and CSF signals **(C,D)** compared to when regression is not performed **(A,B)**. Regression shows little difference in calculated connectivity with subject-specific ROIs **(F)**. Group ROIs also show little difference in calculated connectivity aside from the hippocampus **(E)**.
