# Supplementary figures and images for: Influence of ROI selection on resting state functional connectivity: an individualized approach for resting state fMRI analysis
Source: Front Neurosci. 2015 Aug 11;9:280. doi: 10.3389/fnins.2015.00280 (PMC4531302; doi:10.3389/fnins.2015.00280)

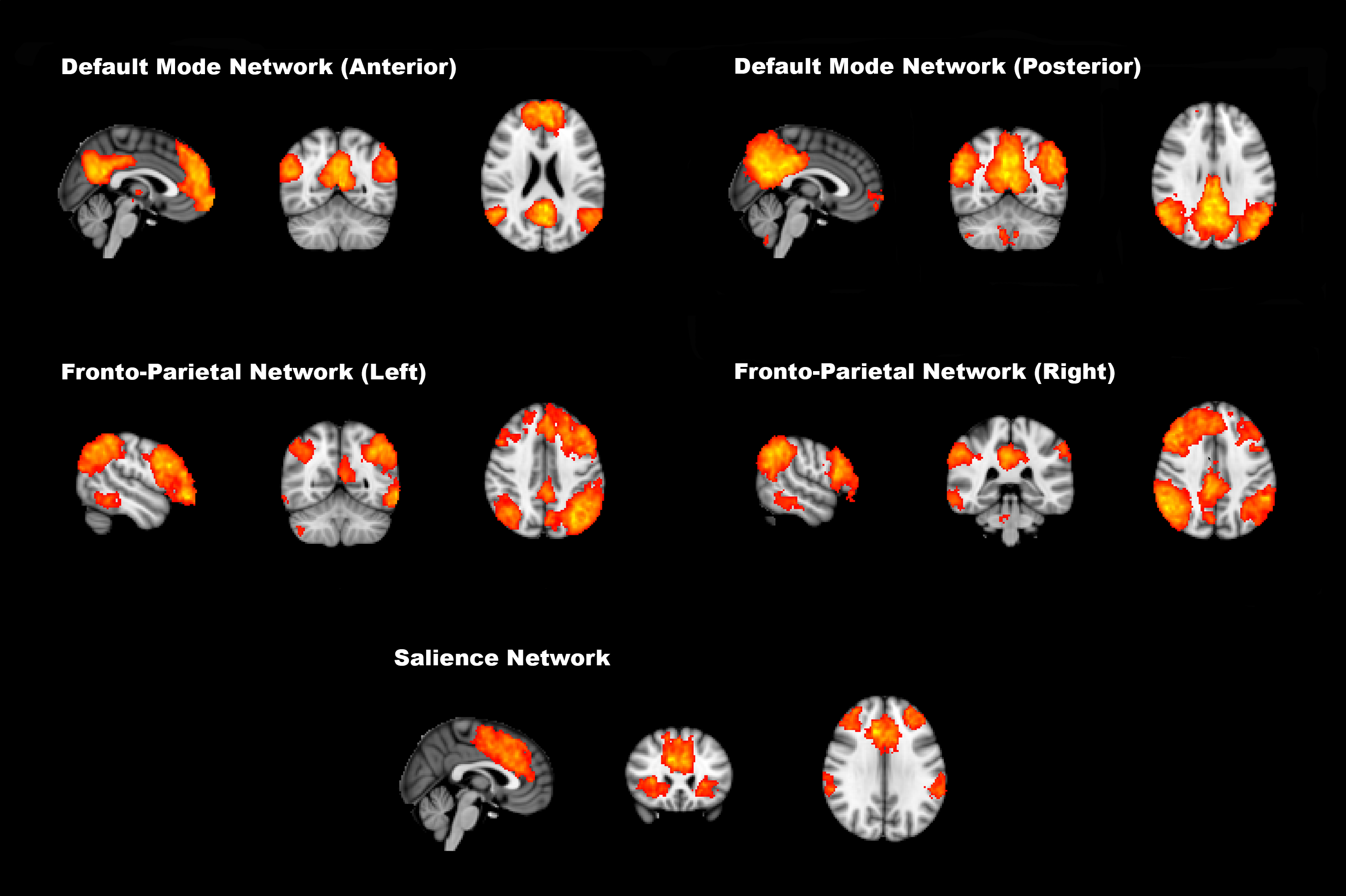

Supplement: Supplementary Figure 1 — Major resting state networks. Networks were obtained using ICA. A total of five networks were selected for subsequent analysis. [file Image1.TIF]

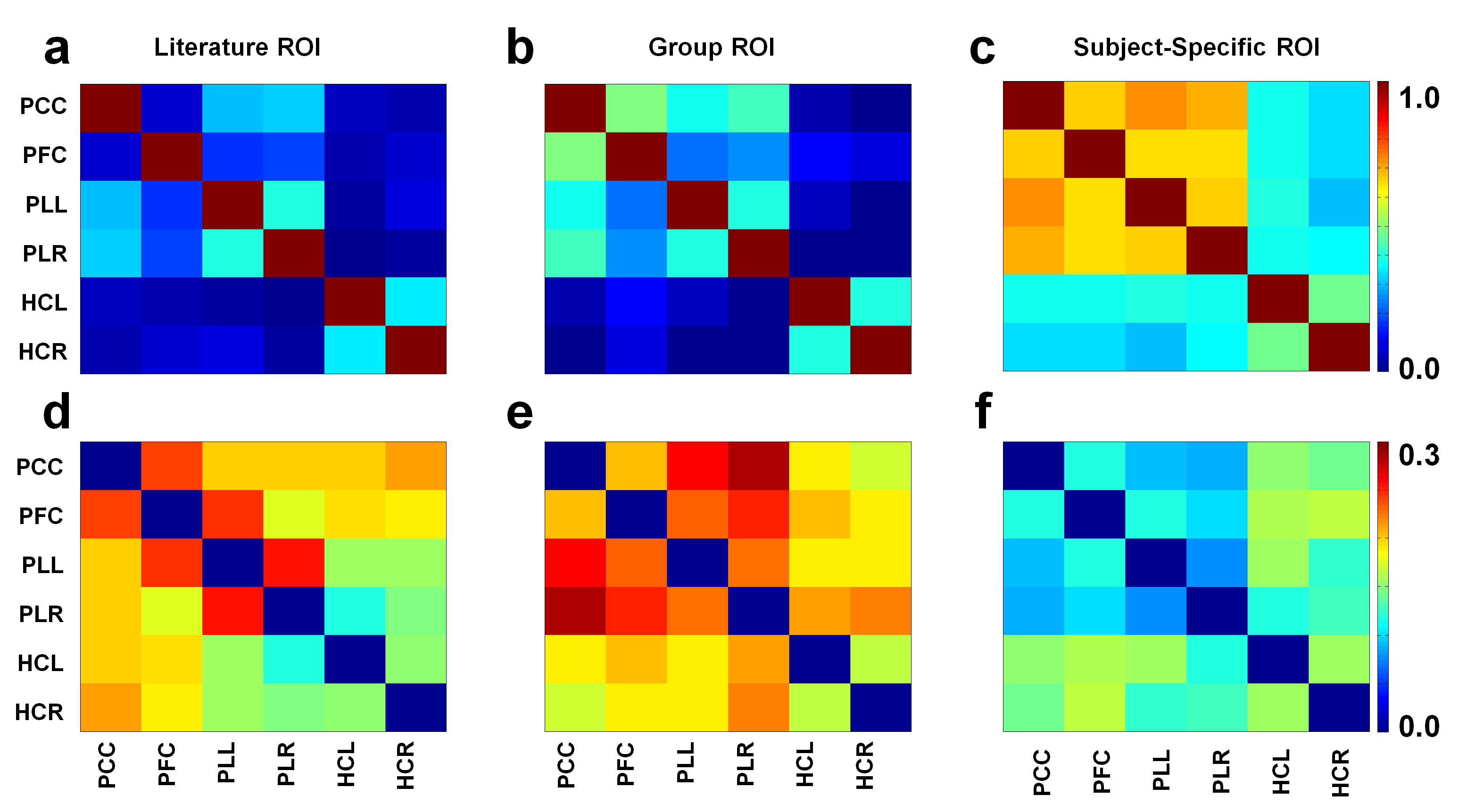

Supplement: Supplementary Figure 2 — Calculated resting state functional connectivity and variance from different ROI seeding methods. Figure shows correlation values for each ROI selection method (A–C) and the variance (D–F). [file Image2.TIF]

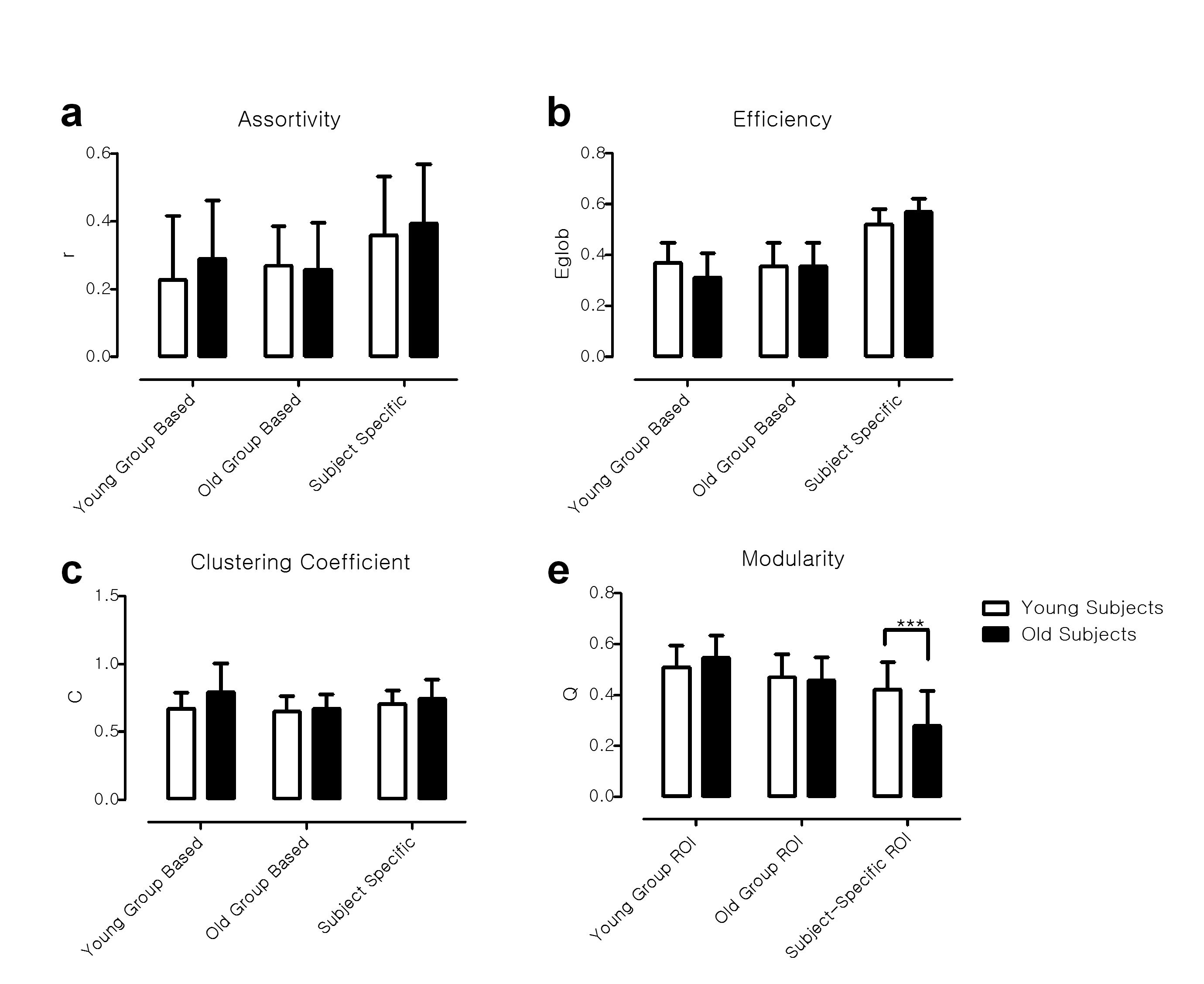

Supplement: Supplementary Figure 3 — Graph theory properties for different ROI selection methods with aging. Properties analyzed include assoritivity (A), efficiency (B), clustering coefficient (C), and modularity (D). Only the modularity obtained when using subject specific ROIs showed any significant differences in aging. [file Image3.TIF]

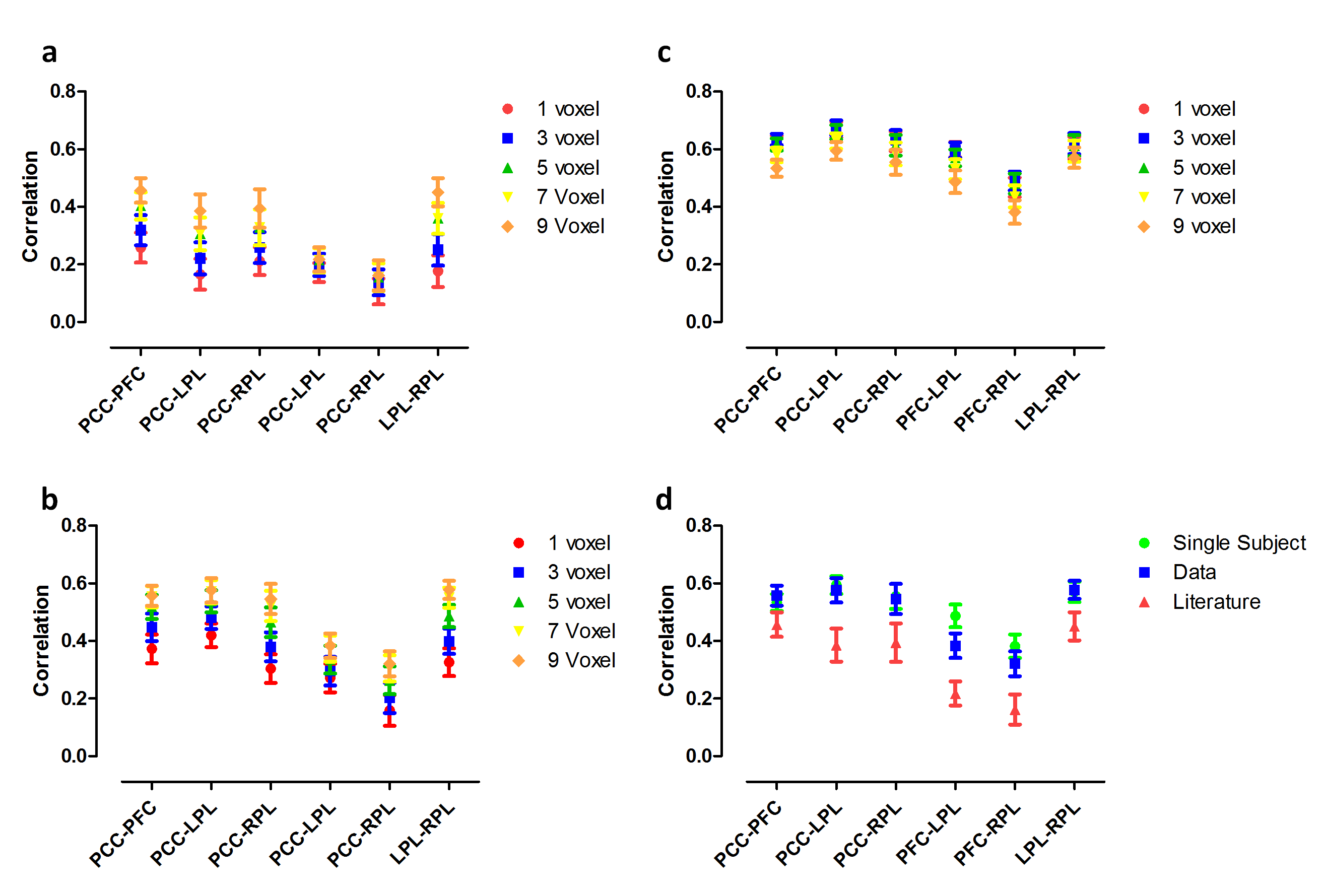

Supplement: Supplementary Figure 4 — Calculated Functional Connectivity with respect to ROI size. Graph shows how functional connectivity between nodes is affected when different ROI sizes are used for node selection when ROIs are selected from literature (A), data (B), and obtained for subject specific ROIs (C). ROIs used are cubic ROIs and label denotes the length of a ROIs edge. Therefore actual ROI size will be n∧3 where n is the length of an edge. (D) Shows the calculated connected between nodes for different ROI selections methods using a 9 Voxel size ROI. PCC, posterior cingulate cortex; PFC, prefrontal cortex; LPL, left parietal lobe; RPL, right parietal lobe. [file Image4.TIF]

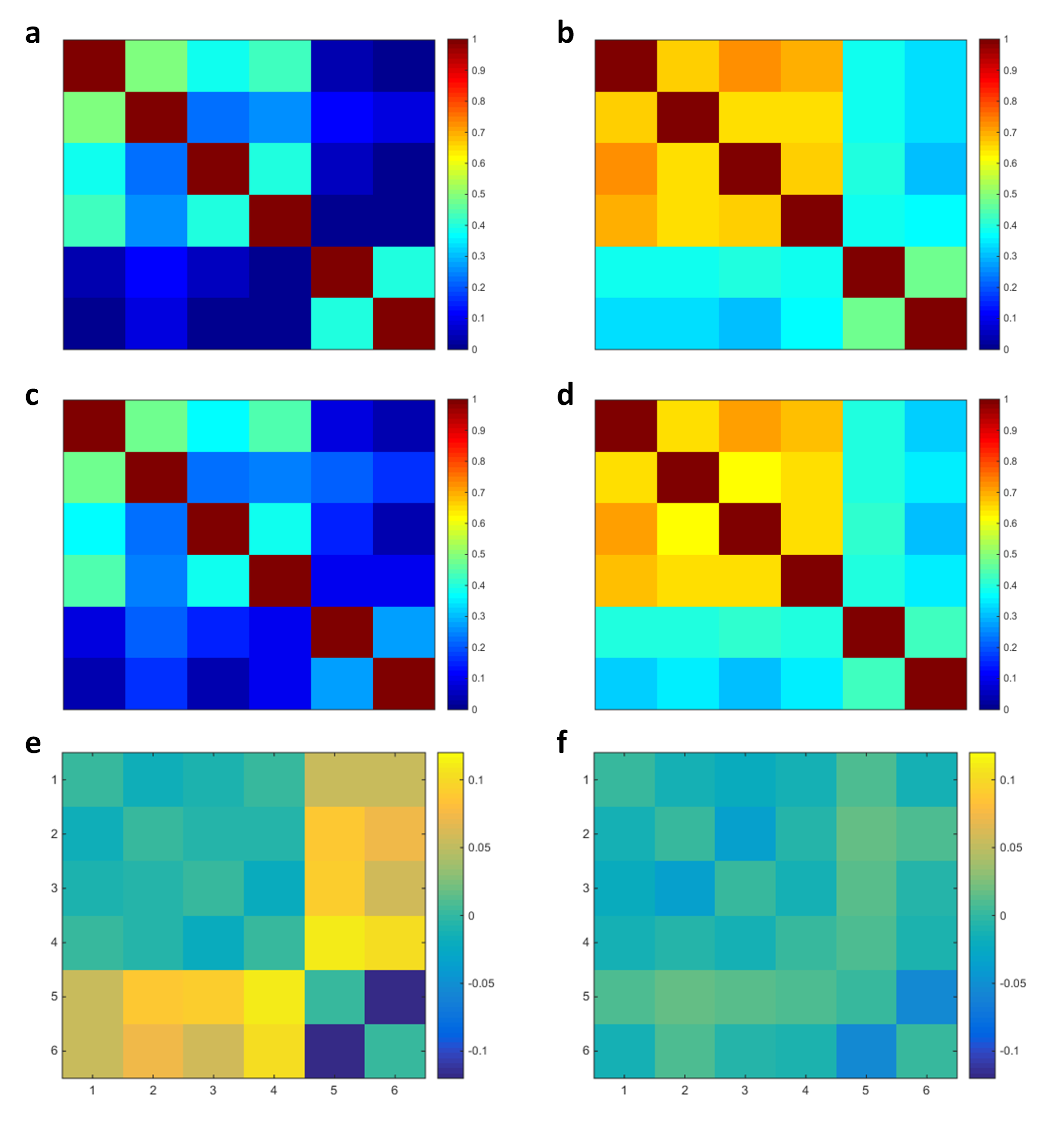

Supplement: Supplementary Figure 5 — Calculated Functional Connectivity with WM and CSF regression. Figure shows average connectivity of the aDMN using group ROIs (A,C) and subject-specific ROIs (B,D) when regressing WM and CSF signals (C,D) compared to when regression is not performed (A,B). Regression shows little difference in calculated connectivity with subject-specific ROIs (F). Group ROIs also show little difference in calculated connectivity aside from the hippocampus (E). [file Image5.TIF]
