# Supplementary material for: Influence of ROI selection on resting state functional connectivity: an individualized approach for resting state fMRI analysis
Source: Front Neurosci. 2015 Aug 11;9:280. doi: 10.3389/fnins.2015.00280 (PMC4531302; doi:10.3389/fnins.2015.00280)
Supplement: Supplementary file 6 [file Table1.DOCX]

**Supplementary Table 1. Mean framewise displacement (FD) for all subjects.** Young subject had slightly lower motion than elderly subjects calculated by FD. Statistical analysis shows that this difference in FD values between young and old subjects is not significant.

|  | Young | Old | p value |
| --- | --- | --- | --- |
| mean FD | 0.0927 | 0.1058 | 0.0564 |
| SD | 0.021 | 0.022 | - |
